# Endotracheal Intubation Using C-MAC Video Laryngoscope vs. Direct Laryngoscope While Wearing Personal Protective Equipment

**DOI:** 10.3390/jpm12101720

**Published:** 2022-10-14

**Authors:** Da Saem Kim, Daun Jeong, Jong Eun Park, Gun Tak Lee, Tae Gun Shin, Hansol Chang, Taerim Kim, Se Uk Lee, Hee Yoon, Won Chul Cha, Yong Jin Sim, Song Yi Park, Sung Yeon Hwang

**Affiliations:** 1 Department of Emergency Medicine, Samsung Medical Center, Sungkyunkwan University School of Medicine, Seoul 06351, Korea; 2 Department of Emergency Medicine, College of Medicine, Kangwon National University, Chuncheon 20341, Korea; 3 Department of Digital Health, Samsung Advanced Institute for Health Science & Technology (SAIHST), Sungkyunkwan University, Seoul 06355, Korea; 4 Health Information and Strategy Center, Samsung Medical Center, Seoul 06351, Korea

**Keywords:** intubation, intratracheal, laryngoscopes, emergency, personal protective equipment, COVID-19

## Abstract

This study sought to determine whether the C-MAC video laryngoscope (VL) performed better than a direct laryngoscope (DL) when attempting endotracheal intubation (ETI) in the emergency department (ED) while wearing personal protective equipment (PPE). This was a retrospective single-center observational study conducted in an academic ED between February 2020 and March 2022. All emergency medical personnel who participated in any ETI procedure were required to wear PPE. The patients were divided into the C-MAC VL group and the DL group based on the device used during the first ETI attempt. The primary outcome measure was the first-pass success (FPS) rate. A multiple logistic regression was used to determine the factors associated with FPS. Of the 756 eligible patients, 650 were assigned to the C-MAC group and 106 to the DL group. The overall FPS rate was 83.5% (n = 631/756). The C-MAC group had a significantly higher FPS rate than the DL group (85.7% vs. 69.8%, *p* < 0.001). In the multivariable logistic regression analysis, C-MAC use was significantly associated with an increased FPS rate (adjusted odds ratio, 2.86; 95% confidence interval, 1.69–4.08; *p* < 0.001). In this study, we found that the FPS rate of ETI was significantly higher when the C-MAC VL was used than when a DL was used by emergency physicians constrained by cumbersome PPE.

## 1. Introduction

The continuing COVID-19 pandemic has had a disastrous effect on healthcare systems worldwide, resulting in significant morbidity and mortality [1]. As the front line of the healthcare system, emergency departments (EDs) have struggled to deal with unprecedented catastrophes [2]. Several efforts have been made to limit the spread of COVID-19 in emergency departments, including the implementation of rigorous infection control measures [3].

SARS-CoV-2 is transmitted primarily through droplets and contact with contaminated surfaces, as well as probably through airborne transmission in certain circumstances, particularly after aerosol-generating procedures [4]. Several airway management techniques, including endotracheal intubation (ETI), bag-mask ventilation, non-invasive positive pressure ventilation, and surgical airways (including cricothyrotomy and tracheostomy), carry a high risk of aerosol generation [5]. As a result, emergency medical personnel (EMP), particularly those involved in airway management procedures, are at risk of viral infection. Given the current COVID-19 pandemic context, it is reasonable for EMP to treat all patients as possibly infected with the disease.

Guidelines from several organizations and experts for airway management have been released during the course of the current pandemic, with the goal of successfully managing COVID-19 patients while minimizing the risk of viral transmission to medical staff [6,7]. These guidelines consistently emphasize the importance of implementing adequate infection control measures, such as wearing sufficient personal protective equipment (PPE) when performing airway management. In addition, the use of a video laryngoscope (VL) has been encouraged from the first attempt at ETI because it allows the operator to perform the procedure more safely than with a direct laryngoscope (DL) by maintaining a greater distance from the patient’s mouth. This recommendation is theoretically plausible. However, it remains unclear whether VLs are a better option than DLs while wearing cumbersome PPE, which can impede the performance of the intubator [8]. Several studies have compared DLs and VLs in a simulation context in which the intubators wore PPE; however, the findings of those studies have conflicted with one another, and clinical evidence is lacking [9,10,11,12].

This study evaluated whether the VL performed better than the DL for ETI while the intubator was wearing PPE in the ED.

## 2. Materials and Methods

### 2.1. Study Design and Setting

This was a single-center, retrospective, observational study conducted from February 2020 to March 2022 in the academic ED of a tertiary university-affiliated referral hospital with more than 70,000 annual ED visits. The semi-rigid stylet is routinely used for endotracheal tube insertion. Rapid sequence intubation is the standard method if a patient resists airway manipulation by medical staff.

**Figure A1:**
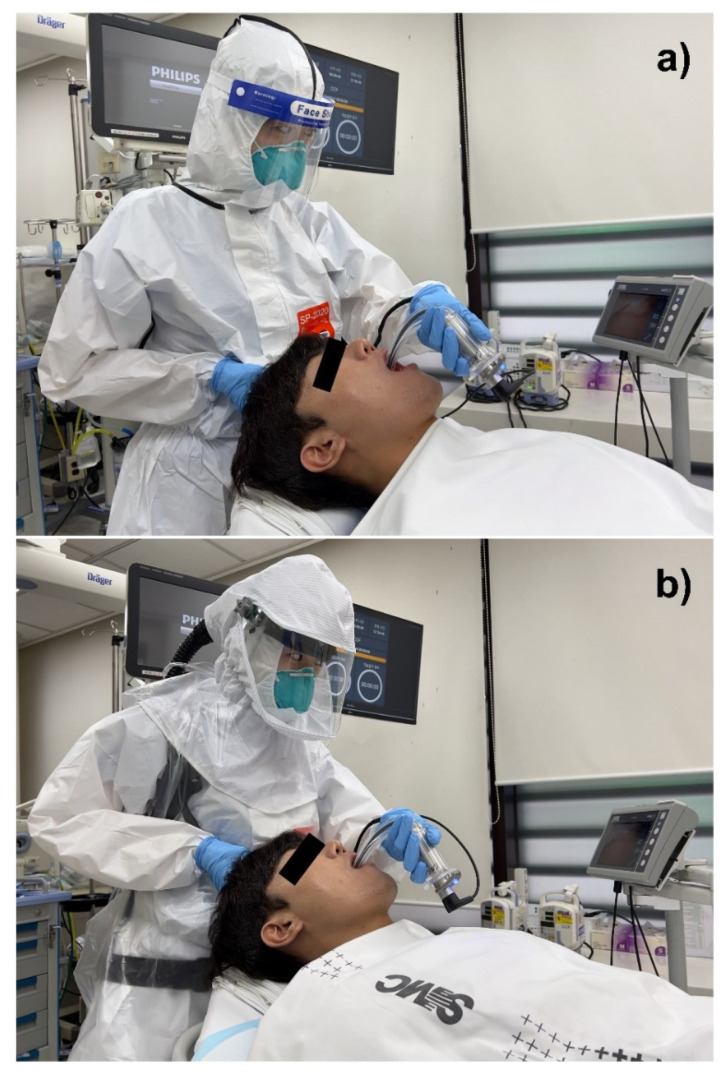
Level of PPE: (**a**) general PPE; (**b**) enhanced PPE. PPE, personal protective equipment. These are staged photographs taken with the consent of the participants.

On 19 January 2020, South Korea reported its first confirmed COVID-19 case [13]. Several infection control measures were implemented in the ED in response to this novel infectious disease [3,14]. Enhanced PPE, a full bodysuit, or at least a waterproof gown, an apron, double gloves, boots, and powered air-purifying respirators (PAPRs) were mandated for all EMP engaging in the ETI procedure for undifferentiated patients with COVID-19-related symptoms (see Appendix A, Figure A1) [14]. The PAPR consists of a loose-fitting hood, a breathing tube, a high-efficiency particulate air filter, and a blower. We use two different PAPRs: A 3M Jupiter Powered Air Turbo with a breathing tube (BT-20 L) and a loose-fitting hood (S-433 L-5) (3M, St. Paul, MN, USA), and an AIR WING III PAPR system with a hood kit (OTOS, Seoul, Korea). General PPE, including a face shield or goggles, was used instead of PAPRs in patients identified as having a low likelihood of COVID-19 infection [14].

### 2.2. Study Population

The primary analysis included adult patients (aged eighteen years or older) who received ETI in the ED. Patients whose ETI used devices other than the C-MAC VL (Karl Storz Endoskope, Tuttlingen, Germany) or a conventional DL on the first attempt were excluded from this study.

### 2.3. Data Collection

The following data were extracted from the institutional airway registry [15] and electronic medical records of our institution: patient age, sex, body mass index (BMI), the level of PPE (enhanced PPE and general PPE), the presence of difficult airway characteristics, the indication for ETI, ETI method (crash approach, rapid sequence intubation, sedative only approach, and other), the intubating device, the drugs given for ETI, the training level of the intubator, the number of ETI attempts, the success of ETI, glottic view (indicated by Cormack–Lehane (C-L) grade), and complications related to ETI. 

### 2.4. Definitions

The patients were divided into two groups based on the device used during the first ETI attempt: the C-MAC group and the DL group. In the C-MAC group, a standard Macintosh-type blade (size 3 or 4) or D-BLADE (Karl Storz Endoskope, Tuttlingen, Germany) was used, whereas in the DL group, a standard Macintosh blade (size 3 or 4) was used. The intubators were divided into three categories based on their training levels: junior residents (first- or second-year residents), senior residents (third- or fourth-year residents), and emergency medicine faculty. The intubator and supervisory staff assessed the presence of difficult airway characteristics for laryngoscopy and ETI, such as obesity, short neck, distorted airway anatomy, facial trauma or anomaly, limited mouth opening (<3 cm), and cervical immobility, based on the patient’s features. The intubator determined the glottic view using the C-L classification method. An ETI attempt was defined as the introduction of a laryngoscope blade into the mouth, regardless of whether an endotracheal tube was inserted. First-pass success (FPS) and multiple attempts were defined as achieving the ETI on the first attempt and three or more ETI attempts, respectively.

### 2.5. Primary and Secondary Outcomes

The primary outcome measure was the FPS rate. The secondary outcomes were glottic view, the rate of multiple attempts, and ETI-related complications. 

### 2.6. Statistical Analysis

Mean with standard deviation (SD) was used to represent continuous variables, and number and percentage is used to describe categorical data. As appropriate, comparisons were performed using Student’s *t*-test, the chi-square test, or Fisher’s exact test. A multiple logistic regression analysis was conducted to determine the factors associated with FPS. Potential confounding variables were selected by clinical plausibility, and the device used for ETI (C-MAC VL or DL) was forced to be included in the model. The following variables are included in the final model: PPE, patient BMI, the presence of difficult airway features, the reason for ETI, and the operator training level. The findings were presented as adjusted odds ratios (aORs) with a confidence interval of 95% (CI). *p*-values less than 0.05 were considered significant for all statistical tests. All statistical analyses were conducted using STATA 15.0 (STATA Corporation, College Station, TX, USA) and R 3.6.1 (R Foundation for Statistical Computing, Vienna, Austria; https://www.r-project.org/, accessed on 1 August 2022).

## 3. Results

### 3.1. Baseline Characteristics of Patients and ETI Procedures

**Figure 1:**
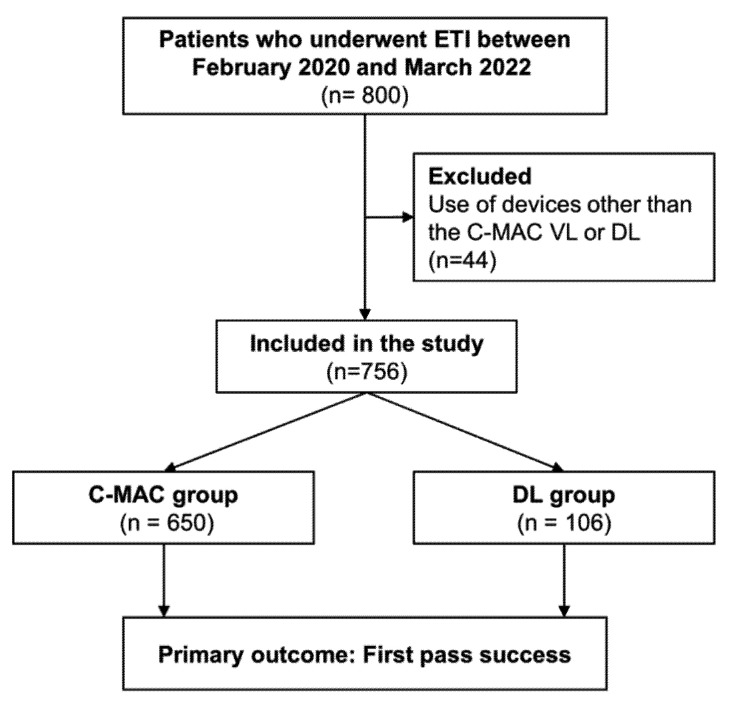
Study flowchart. ETI, endotracheal intubation; C-MAC, C-MAC video laryngoscope; DL, direct laryngoscope.

A total of 800 patients were screened for study eligibility, and 44 were eliminated because they satisfied the exclusion criteria (Figure 1). The remaining 756 patients were divided into two groups: 650 in the C-MAC group and 106 in the DL group. Table 1 summarizes the baseline characteristics of the patients and ETI procedures in the two groups. The mean age of the patients was 66.1 (SD, 15.0) years, and 62.8% were male. The patients’ age, sex, BMI, and intubator level of PPE did not differ significantly between the groups. However, there were significant differences in the indications for ETI between the groups (*p* = 0.001). The most frequently encountered indication in the C-MAC group (45.4%) was respiratory distress, followed by cardiac arrest (35.5%) and airway protection (16.0%). In the DL group, however, the most frequently reported indication was cardiac arrest (45.3%), followed by respiratory distress (25.5%) and airway protection (23.6%). The ETI methods used and the presence of difficult airway characteristics for laryngoscopy did not differ significantly between the groups (all *p* > 0.05). On the first ETI attempt, intubator level and ETI drugs, including sedatives and neuromuscular blocking agents, did not differ significantly between the groups (all *p* > 0.05).

### 3.2. Primary and Secondary Outcomes

**Figure 2:**
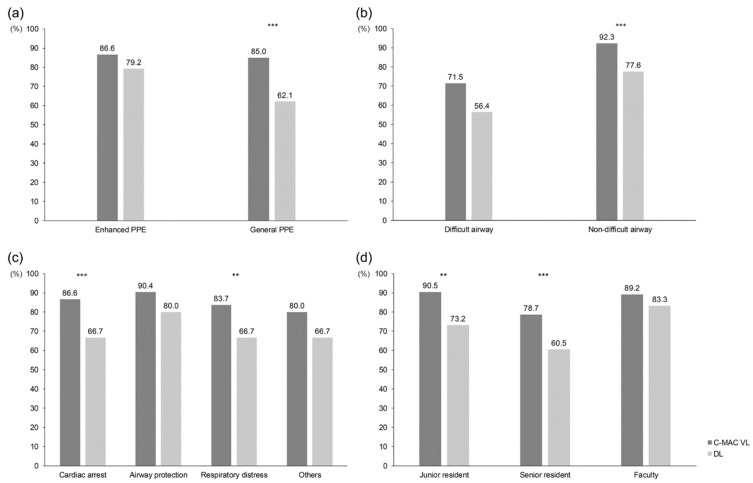
First-pass success rate based on different situations: (**a**) level of PPE; (**b**) anticipated difficult airway; (**c**) indication of ETI; (**d**) level of intubator. PPE, personal protective equipment; C-MAC VL, C-MAC video laryngoscope; DL, direct laryngoscope. ***, *p* < 0.001; **, *p* < 0.05.

Primary and secondary outcomes are presented in Table 2. The overall FPS rate was 83.5% (n = 631/756). The C-MAC group had a significantly higher FPS rate than the DL group (85.7% vs. 69.8%, *p* < 0.001). The FPS rate is presented in Figure 2 in relation to the level of PPE, the presence of difficult airway characteristics, the operator’s level, and ETI indications. Although the FPS rate was significantly higher in the C-MAC group than the DL group when the intubator was wearing general PPE, the FPS rate did not differ between the groups when the intubator was wearing enhanced PPE. In patients who were anticipated to have a difficult airway, the FPS rate did not differ significantly between the two groups; however, the C-MAC VL group had a significantly higher FPS rate in patients who were not anticipated to have a difficult airway. When the intubator was a junior resident (78.7% vs. 60.5%, *p* = 0.01) or a senior resident (90.5% vs. 73.2%, *p* < 0.001), the FPS rate was significantly higher in the C-MAC group than in the DL group, but the rate did not differ between the groups when the intubator was an attending physician (89.2% vs. 83.3%, *p* > 0.05). Difficult glottic visualization, as indicated by a C-L grade of 3 or 4 on the first attempt, was more prevalent in the DL group than in the C-MAC group (19.8% vs. 6.3%, *p* < 0.001). The groups did not differ significantly in terms of ETI-related complications (*p* = 0.704).

### 3.3. Multivariable Analysis for Factors Associated with FPS

C-MAC use was significantly associated with an increased FPS rate in the univariable logistic regression analysis (OR: 2.58; 95% CI: 1.60–4.11; *p* < 0.001) (Table 3). After adjusting for possible confounding variables, this association remained significant (aOR: 2.86; 95% CI: 1.69–4.80; *p* < 0.001). In comparison to cardiac arrest, altered mental status was associated with a higher FPS (aOR 2.85; 95% CI: 1.45–5.88; *p* = 0.003). Senior residents (aOR: 2.51; 95% CI: 1.58–4.02; *p* < 0.001) and attending physicians (aOR: 3.59; 95% CI: 1.67–8.54; *p* = 0.001) had a higher association with FPS than junior residents. The presence of difficult airway characteristics was associated with a lower FPS (aOR: 0.22; 95% CI: 0.14–0.34; *p* < 0.001).

## 4. Discussion

ED physicians face the unique challenge of performing ETIs while wearing cumbersome PPE, which exacerbates the difficulty of the procedure. In this study, we found that the FPS rate of ETI was significantly higher when a C-MAC VL was used instead of a DL by emergency physicians who were constrained by cumbersome PPE. Adjusting for confounding factors in a multivariable analysis did not affect the consistency of that conclusion. Our study is strengthened by the fact that the majority of the ETIs were conducted by emergency physicians who have been actively engaged in clinical practice during the pandemic. In the context of the ongoing COVID-19 pandemic and future preparations for the emergence of new infectious illnesses, it is clinically relevant to evaluate the performance of different ETI devices. Our findings provide additional clinical evidence supporting the use of the C-MAC VL rather than a DL for emergency ETI in a PPE use scenario.

Our findings are consistent with the conclusions of recently published systematic reviews that compared VL with DL. In an extensive systematic review of 222 studies comprising 26,149 adult patients, VLs, regardless of blade design, likely lowered the rates of failed ETI, increased the FPS rate, and provided improved glottic views across various circumstances and patient categories [16]. In addition, in a systematic review with network meta-analyses of 179 studies that ranked VLs for ETI performance relative to DLs in adult patients, VLs generally performed better than DLs for a variety of outcomes, including failed ETI, failed first ETI attempt, failed ETI within two attempts, difficult ETI, glottic visualization, and difficult laryngoscopy [17]. In particular, the C-MAC VL consistently scored the highest across all analyzed outcomes and scenarios. The time required to perform an ETI with a VL and a DL was clinically comparable. However, most of the studies included in those two meta-analyses were conducted in an elective environment without PPE. Therefore, this study, which considered the evidence in the specific context of emergency ETI while the intubator wore PPE, is significant.

In the context of different PPE use situations, the benefits of using VLs rather than a DL have been reported in several previous studies; however, most of those were simulation-based studies, and clinical studies have been lacking. In a simulation study for COVID-19 patients undergoing CPR by paramedics with PPE, Gadek et al. demonstrated that the McGrath VL had significantly better FPS (30% vs. 89%, *p* < 0.001), overall success rate (83% vs. 100%, *p* = 0.002), and median ETI time (34.0 s [29.5–38.5] vs. 24.8 s [21–29], *p* < 0.001) than a DL [9]. In another simulation study, Shin et al. compared Pentax-AWS (AWS) and DL while the intubator wore chemical, biological, radioactive, and nuclear (CBRN) PPE. They found that AWS required less time to complete an ETI than a DL (18.2 s [15.1–22.1] vs. 26.4 s [23.1–35.2], *p* < 0.001) [10]. On the contrary, several studies have failed to provide conclusive evidence that using a VL rather than a DL offers any significant benefits. In a prospective, randomized, crossover manikin study by Yousif et al. comparing a DL, GlideScope Ranger, and King Vision VL used by experienced prehospital providers wearing Level C PPE, successful ETI required significantly more time with the GlideScope Ranger (35.82 s [95% CI, 32.24–39.80 s]) than with the DL (25.69 s [95% CI, 22.42–29.42 s]; *p* < 0.0001) or King Vision (29.87 s [95% CI, 26.08–34.21 s]; *p* = 0.033), which did not differ significantly from each other (*p* = 0.1017) [11]. In addition, Goh et al. compared the McGrath VL and a DL as used by specialized anesthetists wearing PAPR and N95 masks in a randomized controlled trial with 28 patients undergoing elective surgery [12]. In that study, the median times to intubation for VL (61 s [37–63 s]) and DL (41.5 s [37–56 s]) did not differ significantly (*p* = 0.35), and they found no statistically significant differences in the median scores on the intubation difficulty scale or FPS rate. Several factors, including the study design, the study setting and environment, the level of expertise of the intubator, and the outcome measures, could account for the contradictory results of previous studies.

In our study, the use of C-MAC by intubator wearing PPE was associated with a higher FPS rate than the use of a DL. There could be several explanations for these results. First, the large and clear screen of the C-MAC provides a better glottic view than the DL when the intubator’s vision or movement is restricted by PPE such as goggles, a face shield, or a PAPR hood. In a cadaveric study, Taylor et al. compared the DL, McGarth VL, and laryngopharyngeal tube, and most ED residents stated that vision was the most significant barrier to ETI when wearing PPE [18]. Second, it is also possible that the C-MAC VL was more familiar to the intubators than the DL because it was used more frequently during the study period to adhere to pandemic recommendations. The enhanced visibility afforded by the VL could mitigate the challenges of airway visibility caused by wearing PPE, thereby increasing the likelihood of establishing a definitive airway. Third, sharing the screen with a supervisor and receiving guidance on the anatomical structure could have further contributed to the higher FPS rate in the C-MAC group compared with the DL group.

The findings of this study are subject to some limitations. First, this was a single-center study conducted in a tertiary, academic ED; hence, the results might not be applicable in other environments. Because the type and extent of PPE used by our institution might differ from that in other institutions, caution should be used when extending our results to different settings. Second, because this study was retrospective, it was not possible to control for baseline patient characteristics or ETI-related variables. In particular, the device was chosen at the intubator’s discretion. Because this was a retrospective analysis, we were unable to control variables such as intubator preference, expertise, availability of the device, and clinical context, which may impact the choice of device. For these reasons, selection bias was unavoidable. To minimize the impact of selection bias, multivariable analysis was performed in which variables that may influence the FPS rate were adjusted. Third, only the C-MAC VL and DL were compared in this study. A range of VLs are available in the marketplace, and they have considerable heterogeneity in blade design (i.e., channeled vs. non-channeled, hyper-angulated vs. Macintosh-type blade). Because different devices could provide different findings, caution should be used when extending our results to other contexts.

## 5. Conclusions

In circumstances where intubator performance was hindered by cumbersome PPE, the FPS rate of ETI was significantly higher when the intubator used C-MAC VL rather than DL. When the intubator is wearing PPE, our results give clinical support for the use of the C-MAC VL over a DL.

## Figures and Tables

**Table 1:** Baseline characteristics of patients and endotracheal intubation procedures.

	Total (n = 756)	C-MAC Group (n = 650)	DL Group (n = 106)	*p*-Value
Patient age (years)	66.1 ± 15.0	66.3 ± 15.0	65.1 ± 15.2	0.445
Patient sex (male)	475 (62.8)	413 (63.5)	62 (58.5)	0.374
Patient BMI (kg/m^2^)	22.8 ± 4.0	22.9 ± 4.0	22.3 ± 3.9	0.102
Patient BMI				0.194
Normal weight (18.5–24.9 kg/m^2^)	484 (64.0)	418 (64.3)	66 (62.3)	
Underweight (<18.5 kg/m^2^)	92 (12.2)	73 (11.2)	19 (17.9)	
Pre-obese (25.0–29.9 kg/m^2^)	140 (18.5)	125 (19.2)	15 (14.2)	
Obese (≥30.0 kg/m^2^)	40 (5.3)	34 (5.2)	6 (5.7)	
PPE level				0.737
General	425 (56.2)	367 (56.5)	58 (54.7)	
Enhanced	331 (43.8)	283 (43.5)	48 (45.3)	
Anticipated difficult airway	246 (32.5)	207 (31.8)	39 (36.8)	0.370
Intubation indication				0.001
Cardiac arrest	279 (36.9)	231 (35.5)	48 (45.3)	
Airway protection	129 (17.1)	104 (16.0)	25 (23.6)	
Respiratory distress	322 (42.6)	295 (45.4)	27 (25.5)	
Other	26 (3.4)	20 (3.1)	6 (5.7)	
Method for ETI				0.135
Crash approach *	284 (37.6)	235 (36.2)	49 (46.2)	
RSI	395 (52.2)	348 (53.5)	47 (44.3)	
Other	77 (10.2)	67 (10.3)	10 (9.4)	
Level of intubator †				0.736
Junior resident	296 (39.2)	258 (39.7)	38 (35.8)	
Senior resident	383 (50.7)	327 (50.3)	56 (52.8)	
Faculty	77 (10.2)	65 (10.0)	12 (11.3)	
Sedatives (n = 434)				0.719
Ketamine	117 (27.0)	106 (27.5)	11 (22.4)	
Etomidate	289 (66.6)	253 (65.7)	36 (73.5)	
Other ‡	27 (6.2)	25 (6.5)	2 (4.1)	
NMBAs (n = 404)				0.768
Succinylcholine	146 (36.1)	130 (36.6)	16 (32.7)	
Rocuronium	246 (60.9)	214 (60.3)	32 (65.3)	
Other §	12 (3.0)	11 (3.1)	1 (2.0)	

The data are presented as or number (%). * Crash approach: This was used for unconscious, unresponsive patients who were not expected to be resistant to laryngoscopy and needed immediate airway security. † Junior residents are first- and second-year residents. Senior residents are third- and fourth-year residents. ‡ Lorazepam and midazolam. § Vecuronium and cisatracurium. Abbreviations: VL, video laryngoscope; DL, direct laryngoscope; BMI, body mass index; ETI, endotracheal intubation; RSI, rapid sequence intubation; NMBAs, neuromuscular blocking agents.

**Table 2:** Primary and secondary outcomes.

	Total (n = 756)	C-MAC Group (n = 650)	DL Group (n = 106)	*p*-Value
**Success rate**				
First-pass success rate	631 (83.5)	557 (85.7)	74 (69.8)	<0.001
Multiple attempts *	37 (4.9)	27 (4.2)	10 (9.4)	0.036
**Glottic view**				
C-L grade III or IV, (%)	62 (8.2)	41 (6.3)	21 (19.8)	<0.001
**Complications**				
Any complications	120 (15.9)	105 (16.2)	15 (14.2)	0.601
Esophageal intubation	8 (1.1)	5 (0.8)	3 (2.8)	0.054
Unrecognized EI †	0 (0.0)	0 (0.0)	0 (0.0)	-
Dental injury	3 (0.4)	2 (0.3)	1 (0.9)	0.334
Post-intubation hypotension ‡	64 (8.5)	56 (8.6)	8 (7.5)	0.714
Post-intubation hypoxemia ‡	23 (3.0)	19 (2.9)	4 (3.8)	0.636
Vomiting ‡	1 (0.1)	1 (0.2)	0 (0.0)	0.686
Agitation ‡	21 (2.8)	20 (3.1)	1 (0.9)	0.215
Cardiac arrest	14 (1.9)	13 (2.0)	1 (0.9)	0.454

The data are presented as number (%). * Multiple attempts are defined as three or more intubation attempts. † Unrecognized EI was defined as esophageal intubation found after the patient’s condition worsened. ‡ These variables were observed in patients without cardiac arrest. BMI, body mass index; C-L grade, Cormack and Lehane grade; EI, esophageal intubation.

**Table 3:** Univariable and multivariable analyses to identify the factors associated with FPS.

	Univariable		Multivariable	
Parameter	OR (95% CI)	*p*-Value	OR (95% CI)	*p*-Value
**Device, C-MAC VL (vs. DL)**	2.58 (1.60–4.11)	<0.001	2.81 (1.68–4.71)	<0.001
**PPE**				
General PPE	(ref)		(ref)	
Enhanced PPE	1.30 (0.88–1.93)	0.185	1.25 (0.81–1.93)	0.312
**BMI,** kg/m^2^				
Normal weight (18.5–24.9)				
Underweight (<18.5)	0.79 (0.44–1.43)	0.438	0.88 0.46–1.70)	0.703
Pre-obese (25.0–29.9)	0.52 (0.33–0.82)	0.005	0.60 (0.37–1.00)	0.050
Obese (≥30.0)	0.94 (0.38–2.33)	0.897	2.06 (0.78–5.45)	0.146
**Anticipated difficult airway**	0.24 (0.16–0.35)	<0.001	0.22 (0.14–0.33)	<0.001
**Intubation indication**				
Cardiac arrest	(ref)		(ref)	
Non-cardiac arrest	1.04 (0.70–1.54)	0.860	1.68 (1.04–2.71)	0.033
**Level of intubator**				
Junior resident (1st, 2nd)	(ref)		(ref)	
Senior resident (3rd, 4th)	2.26 (1.51–3.43)	<0.001	2.29 (1.43–3.66)	0.001
EM Faculty	2.34 (1.16–5.24)	0.025	3.43 (1.51–7.77)	0.003

FPS, first-pass success; OR, odds ratio; CI, confidence interval; C-MAC VL, C-MAC video laryngoscope; DL, direct laryngoscope; PPE, personal protective equipment; BMI, body mass index.

## Data Availability

The datasets used in this work are available upon reasonable request from the corresponding author and are not publicly available.
